# Comments on Bozzatello et al. Supplementation with Omega-3 Fatty Acids in Psychiatric Disorders: A Review of Literature Data. *J. Clin. Med.* 2016, *5*, 67

**DOI:** 10.3390/jcm5080069

**Published:** 2016-08-03

**Authors:** Gregor Berger

**Affiliations:** Department of Child and Adolescent Psychiatry and Psychotherapy, Outpatient Clinics and Specialized Care, Emergency Services, University Hospital of Psychiatry Zurich, Neumünsterallee 3, P.O. Box 1482, 8032 Zurich, Switzerland; Gregor.Berger@puk.zh.ch; Tel.: +41-43-499-26-71

Paola Bozzatello et al. [1] have done a comprehensive qualitative review of the potential use of long-chain polyunsaturated fatty acids in the prevention and treatment of mental disorders. The number of placebo-controlled trials across a range of mental disorders has surged substantially over the last two decades; however, only very few well-designed large scale trials have been performed. Therefore, even in conditions like schizophrenia, depression or attention deficit and hyperactivity disorders (ADHD) where most omega-3 fatty acid RCTs have been performed, no final conclusions regarding the use of omega-3 fatty acids can be drawn yet. One of the key problems of systematic reviews investigating the use of omega-3 fatty acids in mental disorders is that they have integrated quite diverse phenotypic groups, e.g., the use of omega-3 fatty acids in controlled treatment trials that also assessed depressive symptoms included the following different conditions:

Primary diagnosis of adult major depressive disorders (MDD) [2,3,4,5,6,7,8,9,10,11,12,13,14,15,16]
Depressive episodes in bipolar affective disorders [17,18,19,20,21,22,23]
Depression during or post pregnancy (postpartum depression) [24,25,26]
Depression in non-MDD mood disorders (e.g., premenstrual syndrome, dysthymia) [27,28,29,30,31,32]
Depression in other psychiatric conditions (e.g., borderline PD, self-harm, OCD) [33,34,35,36,37]
Depression in established schizophrenia [2,38]
Depression in Alzheimer’s dementia/mild cognitive impairment [13,39,40]
Depression in Parkinson disease [41]
Depression in medical conditions (cerebro-vascular and metabolic diseases or cancer) [42,43,44,45,46]
Depressive symptoms in healthy individuals [47,48,49,50,51,52]

Several meta-analytic reviews have tried to integrate the above-mentioned very heterogeneous controlled treatment trials investigating the effects of omega-3 fatty acids on mood symptoms [53,54,55,56,57,58,59]. Most meta-analysis including RCTs investigating depressive syndromes confirmed a statistical significant effect in favour of omega-3 fatty acids with minimal to moderate effect sizes depending on the selection of studies (except of one meta-analysis [57]). Effect sizes in favour of omega-3 fatty acids [60] are larger if RCTs are selected based on (1) a EPA/DHA ratio >60% of the overall omega-3 fatty acid content [54,56] and (2) only RCTs with a primary diagnosis of MDD are included [54,59]. To our knowledge, only one pilot RCT (*n* = 20) in children with a mean age of 10 was performed [61]. Martins et al.’s [54] meta-analysis including RCTs with primary and secondary MDD found a significant overall SMD = −0.291 in favour of omega-3 fatty acids, but also detected a marked study heterogeneity and evidence for publication bias. A more recent meta-analysis by Sublette et al. [56] only including primary MDD RCTs dichotomized according to a EPA/DHA ratio >60% of the overall omega-3 fatty acids content found a moderate effect size (SMD = 0.558) with negligible contribution of random effects or heteroscedasticity. Bloch and Hannestadl’s meta-analysis [57] including studies with mildly depressed individuals not meeting criteria for clinical depression could not replicate previous meta-analyses; however, a sub-analysis restricted to moderate to marked depression confirmed an SMD of 0.42 in favour of omega-3 fatty acid treatment. It is likely that a single study by Rogers et al. [27] investigating the effects of omega-3 fatty acids on mild depressive symptoms in a large non-clinical population was responsible for the negative overall outcome as the Rogers study accounted for 31.7% of the overall weight in this particular meta-analysis [62]. Grosso et al. [59] found a SMD = 0.56 for primary MDD, an SMD = 0.22 for non-primary MDD, and an overall SMD = 0.38 in favour of omega-3 fatty acid compared to placebo treatment. The above mentioned meta-analyses suggest that mainly the use of EPA rather than DHA rich formulations are responsible for the clinical efficacy of omega-3 fatty acids. Unexpected is the finding that the use of purified or DHA-enriched oils is not successful in treating depression, postnatal depression or OCD [4,63,64]. This finding is in contrast to the greater face validity of DHA, which is the major brain omega-3 fatty acids and which is lower in brain tissue of depressed suicide victims [65].

Two RCTs encompassing a large proportion of patients with refractory depression highlight the potential use of EPA-enriched omega-3 fatty acids as an augmentation treatment of antidepressants (potentially via an increase in membrane fluidity) [2,3]. Two RCTs in populations without a primary MDD provide evidence of an association between inflammation and omega-3 fatty acids response: (1) A placebo-controlled trial investigating the positive effects of omega-3 fatty acids on depressive symptoms and chronic inflammation in haemodialysis patients [66]; and (2) a study [67] that found a preventive effect of EPA against the development of depressive symptoms in IFN-alpha-treated hepatitis C virus carriers (associated with a very high risk of drug-induced depressive symptoms). The latter two studies suggest that omega-3 fatty acids rich in EPA may modulate its antidepressant properties via immune-modulatory strategies, which is of interest in the light of more recent models of the underlying pathophysiology of a range of mental disorders [68].

A whole range of RCTs have also been performed in schizophrenia and related disorders. A study in first episode psychosis adolescents demonstrates that omega-3 fatty acids augmentation treatment of antipsychotic medication may result in a better tolerability (less EPS, less sexual side effects) and faster response to antipsychotic medication; however, at the end of the three month treatment period, there was no difference in treatment effects on all primary outcome measures between active and placebo [69]. The final outcome of this study is in line with a meta-analysis of Dr. Fusar-Poli and the author of this commentary coming to the conclusion that omega-3 fatty acids in established (but not prodromal) schizophrenia have no or only minor additional efficacy compared to currently available treatments (also not on depressive symptoms in schizophrenia) [70], but may have some beneficial effects in tertiary prevention.

The use of omega-3 fatty acids in primary (indicated) prevention of mental disorders in general may be a separate important area of omega-3 fatty acid research that goes beyond the schizophrenia prodrome, and is an avenue yet to be further explored. A pilot RCT in 81 adolescents at ultra-high risk (UHR) for developing a psychotic disorder (mean age 16.4) compared 1.2 g of an EPA-enriched omega-3 fatty acids oil as a sole agent with a placebo oil in a double blind fashion [71]. A total of 27.5% in the placebo group progressed towards a first psychotic episode compared to only 4.9% in the omega-3 fatty acids group. A recent multinational multicentre study (the NEURAPRO study [72]) including over 300 UHR adolescents tried to replicate this promising pilot study. However, the key problem of the replication study is that the overall transition rate of the study after one year was as low as 10.5% and the compliance rate of 43% was very poor so that a reasonable conclusion at this stage of data analysis is not really appropriate [73]. Another multinational multi-centre omega-3 RCT in prodromal schizophrenia will start in the near future (the PURPOSE trial). Bozzatello et al. [1] discussed the latter studies in the schizophrenia section. However, as only 10% to 20% of URH adolescents will progress to a first psychotic episode within one year and only about half these first episode cases actually meet criteria for core schizophrenia, it is probably not correct to discuss URH studies within the schizophrenia section. It may be much more appropriate to investigate neuroprotective interventions like omega-3 fatty acids not solely within a schizophrenia concept, but much more in the light of brain developmental factors and biological relevant markers, such as the inflammatory markers, the omega-3 index or markers of neuronal damage (e.g., TNF-beta).

The above mentioned small pilot omega-3 RCT in prepubertal children with childhood-onset depression shows a very large effect size (SMD = 1.2) [61]. Also the pilot study in UHR adolescents for psychosis [71] with a mean age of 16.1 showed large effect sizes. Furthermore, most omega-3 fatty acids RCTs in children were done in ADHD and show a beneficial effect. Bloch et al.’s meta-analysis [74] including 699 ADHD children of ten RCTs between 7 and 12 years found a beneficial effect in favour of omega-3 fatty acids with a SMD of 0.31 with no evidence of publication bias and a significant dose dependency; RCTs using a daily dose of 500 to 750mg EPA were the most effective ones [75]. Even so, the effect size of stimulant treatment with methylphenidate, dexamphetamines or atomotexine is still two to four times stronger compared to omega-3 fatty acids alone, the positive findings of omega-3 fatty acid studies in children is suggestive that in particular the developing brain may benefit from omega-3 fatty acids. Future research has to address the question if subgroups of children with mental disorders may benefit more from benign interventions like omega-3 fatty acids compared to adults with established (end stage) mental disorders.

For all other mental disorders and associated conditions only limited evidence exists to promote or refute the use of Omega-3 fatty acids in daily clinical care. As outlined by Bozzatello et al. [1], there is some evidence that omega-3 fatty acids augmentation may have some beneficial effects in bipolar affective disorders [17], in particular against depressive symptoms [19,76]. Furthermore, EPA-enriched omega-3 fatty acids may also attenuate impulsivity in patients with Borderline Personality Disorder [35,77] and incarcerated young males [78]. The latter findings may be of particular importance for male pediatric MDD individuals that sometimes present with impulsive and aggressive behaviour rather than sadness [25]. A recently published trial in adolescents with conduct disorders highlights the importance to implement long study durations (e.g., one year) to be able to demonstrate potential positive effects of omega-3 fatty acids on difficult to treat behavioural traits [79]. Worth mentioning is a recent RCT in premenstrual syndrome (PMS) showing some beneficial effects on depression, nervousness, anxiety, lack of concentration and a reduction of somatic symptoms such as bloating, headaches and breast tenderness [32]. These studies need replication, but point towards important phenotypic features that may benefit from omega-3 fatty acids, such as impulsivity. We may consider more complex trial designs to address the questions if omega-3 fatty acids may be of importance across a whole range of mental disorders, in particular in childhood and adolescents and for certain phenotypic features.

The underlying mechanisms of the potential preventive and therapeutic actions of omega-3 fatty acids against mental disorder are still unclear. Preclinical and clinical data point towards several mechanisms most likely acting in concert [80]. Some of them might be more responsible for short-term, other for postulated long-term effects of omega-3 fatty acids. There is some preclinical evidence that Omega-3 fatty acids may modulate the HPA-axis that is suggested to play a role in a range of mental disorders [81]. Omega-3 fatty acids have shown to attenuate stress-related changes in animal models with depressive features [82,83,84,85] as well as in humans [86,87,88]. Furthermore, Omega-3 fatty acids may influence myelination and synaptic pruning, important processes for normal pubertal brain development. The regulation of PUFA metabolism is crucial for both processes [89,90]. Of particular interest is a preclinical study investigating cognition and behaviour across different developmental stages. Omega-3 fatty acids deficient diets across consecutive generations produced a modality-selective and task-dependent impairment in cognitive and motivated behaviour in adolescence distinct from the deficits observed in adults [91,92]. Omega-3 fatty acids attenuate such depression-like animal behaviours during critical periods of brain development [93]. Furthermore, the FADS haplotype determining LC-PUFAs availability and concentrations in white matter (WM) showed age-related WM differences in humans (significant age × genotype interactions, *p*(corrected) < 0.05). PUFA metabolism is therefore likely to play a role in disorders of neurodevelopmental origin [94]. Animal models with structural hippocampal alterations with depression-like and anxiety-like behaviours [95,96] provide evidence that omega-3 fatty acids have a preventive and neurotrophic effect against hippocampal changes [97,98]. Omega-3 fatty acids enhance hippocampal cell viability and are able to protect hippocampal cells from stress-related damage [99]. Monoaminergic transmitter systems are proposed to be involved in the pathogenesis of many mental disorders. Animal experiments of omega-3 fatty acids deprived rats provide evidence for an increase in serotonin 2 (5-HT2) and a decrease in dopamine 2 (D2) receptor density in the frontal cortex, as well as an increased serotonin turnover in the prefrontal cortex and decreased midbrain tryptophan hydroxylase-2 expression [100,101,102,103,104,105,106]. In humans, omege-3 intake is associated with an increase in cerebrospinal fluid 5-HIAA release [107,108]. Several lines of evidence support that Omega-3 fatty acids have immune-modulatory, anti-inflammatory and pro-resolving properties [109], e.g., via the modulation of pro-inflammatory omega-6, the promotion of proresolvins, neuroprotectins and anti-inflammatory mediators [110,111,112]. Omega-3 fatty acids seem to induce protective in vivo brain mechanisms against oxidative stress. Ethyl-EPA supplementation is associated with a marked increase of glutathione, a strong intracellular antioxidant using proton magnetic resonance spectroscopy in patients with a first-episode psychosis [113]. Another group found similar effects in older patients at risk for depression [114]. Some evidence regarding the measurement of glutathione in peripheral blood is also suggestive that omega-3 fatty acids may support the antioxidative defence system in individuals at ultra-high risk for psychosis [115]. Finally, a decrease in membrane fluidity can affect the rotation and diffusion of proteins and other bio-molecules within the membrane, thereby affecting the functions of these molecules and processes. An increase in membrane fluidity results in a more flexible membrane and facilitates transmission (e.g., in the retina) [116]. In vivo imaging techniques such as diffusion tensor imaging could demonstrate that omega-3 fatty acids are closely linked to PUFA metabolism [94]. The effect of omega-3 fatty acids on membrane structure [117] may contribute to its clinical effects, in particular in augmentation studies. T2-relaxation time normalizes under the influence omega-3 fatty acids potentially being a signifier of normalization in membrane structure [118]. Future studies should therefore address if particular markers, such as low baseline levels of omega-3 fatty acids, increased inflammatory mediators, markers of intact myelination (e.g., measured with DTI), or a functional glia-neuronal interface (e.g., measured with MRS) may serve as predictors of omega-3 fatty acid response, in particular in children and adolescents in the early course of disorders [119]. Bozzatello et al.’s [1] qualitative and comprehensive review is a contribution to this endeavour and highly recommended to interested readers.
